# Activity and Stability Studies of Verbascoside, a Novel Antioxidant, in Dermo-Cosmetic and Pharmaceutical Topical Formulations 

**DOI:** 10.3390/molecules16087068

**Published:** 2011-08-18

**Authors:** Silvia Vertuani, Erika Beghelli, Emanuela Scalambra, Gemma Malisardi, Stefano Copetti, Roberto Dal Toso, Anna Baldisserotto, Stefano Manfredini

**Affiliations:** 1 Department of Pharmaceutical Sciences, University of Ferrara, Via Fossato di Mortara 17-19, 44121 Ferrara, Italy; 2 Ambrosialab, University of Ferrara, Via Mortara 171, 44121 Ferrara, Italy; 3 Biotechnological Research Institute (I.R.B.), Via Lago di Tovel, 7, 36077 Altavilla Vicentina (VI), Italy

**Keywords:** verbascoside, VPP, antioxidant activity, PCL, HPLC analysis, stability in dermocosmetic formulations, suppositories, sustainability, meristematic cells

## Abstract

We here report the results of our investigations carried out on verbascoside, a phenylpropanoid glycoside known for its antioxidant, anti-inflammatory and photoprotective actions. Verbascoside was obtained from *Buddleia davidii* meristematic cells, obtained in turn using a sustainable biotechnology platform which employs an *in vitro* plant cell culture technology. Verbascoside was first investigated to assess the behaviour of the active ingredient in solution or in finished preparations, in view of its potential topical use, especially in skin protection. Stability studies were performed by HPLC, and a PCL assay was adopted to determine the radical scavenging activity toward superoxide anion. The high hydrophilic character of verbascoside, suggested in a somewhat limited range of possible applications, leading us to explore its derivatization to obtain the semi-synthetic derivative VPP, an acyl derivative of verbascoside, with an improved range of applications due to its lower hydrophilic profile. Alone, VPP revealed increased antioxidant activity, both as an active ingredient and in dermocosmetic preparations. Stability studies showed a greater stability of VPP in lipophilic vehicles, whereas the parent verbascoside proved more stable in an O/W emulsions. Verbascoside was also stable in suppositories, an interesting pharmaceutical form for possible applications in treatment of inflammation of the intestinal mucosa.

## 1. Introduction

**Figure 1:**
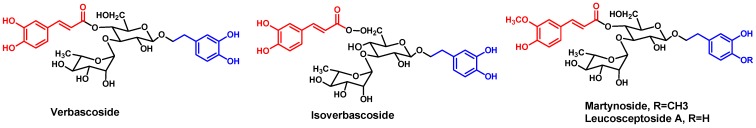
Chemical structure of *Buddleia davidii* phenylpropanoids: verbascoside, isoverbascoside, martynoside and leucosceptoside A. Cinnamic and phenylethanolic moieties are highlighted by red and blue colour respectively.

Oxidative stress is considered to play an essential role in the pathogenesis of aging and degenerative diseases [1,2]. It results in oxidative alteration of biological macromolecules such as lipids, proteins and nucleic acids. In order to cope with an excess of free radicals produced upon oxidative stress, human body has developed sophisticated mechanisms for maintaining redox homeostasis. These protective mechanisms include scavenging or detoxification of reactive oxygen species (ROS), blocking ROS production, sequestration of transition metals, as well as enzymatic and non-enzymatic antioxidant defences that are endogenous [3] when produced in the body or exogenous if supplied with the diet. The skin, a highly metabolic tissue that possesses the largest surface area in the body, represents the major target for free radical damage. Particularly, it is well known that free radicals and ROS are involved in the mechanisms leading to cutaneous damages, such as early ageing, inflammatory disorders and skin cancers. Over the last decade antioxidants have been proposed as functional ingredients for anti-aging preparations, and to prevent and modulate oxidative skin damage. Market demands, increasingly oriented towards natural antioxidants, have also fostered the research and development of innovative cosmeceutical ingredients of vegetable origin with radical-scavenger activity. In this context and prompted by our continuous interest in this field, we have recently investigated extracts of butterfly bush (*Buddleja davidii*), a shrub in the Buddlejaceae family widely used as an ornamental plant and known in traditional medicine for their wound healing, anti-inflammatory, diuretic, anti-allergic detergent, antiviral and antibacterial properties. The *Buddleja davidii* extract used in the present investigation derives from a biotechnology platform, which employs *in vitro* plant cell culture technology [4]. This biotechnology method allows one to obtain plant extracts enriched in the secondary metabolites, at concentrations that cannot be obtained by traditional methods (*i.e.*, extraction from wild plants, synthesis). The principal components of the *Buddleia davidii* extracts, were identified as phenylpropanoid derivatives such as verbascoside, isoverbascoside, leucosceptoside A and martynoside (Figure 1), together with a further component constituted mainly by oligo- and polysaccharides, proteins and lipids (chromophore-free fraction).

Verbascoside is the most abundant of these chemical substances and may be found in very high concentrations (approximately 80%) in *Buddleja davidii* cell cultures. The biological properties of verbascoside, also known as acteoside, have been described in the literature and comprise a wide spectrum of activities, including antioxidant, anti-inflammatory, photoprotective and chelating actions.

In particular, a significant antioxidant effect of verbascoside has been recently documented by Aleo *et al*. in an experimental study comparing numerous natural antioxidant substances using various method of determination [5,6,7,8]. Furthermore the anti-inflammatory activity of verbascoside has been confirmed by an *in vitro* test performed on cell cultures of primary human keratinocytes [7], in which verbascoside was able to significantly reduce, in a dose-dependent manner, the release of pro-inflammatory chemokines. This study has also demonstrated that verbascoside promotes skin repair and ameliorates skin inflammation due to its ROS scavenging, antioxidant, iron chelating, and glutathione transferase (GST) activity inducing properties [9,10]. An *in vivo* study, conducted on inflammation of the intestinal mucosa, demonstrated that verbascoside is able to inhibit the activation of pro-inflammatory proteins and consequently the enzymatic activity of matrix metalloproteinases, the latter also being involved in skin aging phenomena. The results of this study suggested that verbascoside functions as an intracellular radical scavenger and thus reduces the microscopic and macroscopic signs of colitis in the rat. Therefore, administration of verbascoside may be beneficial for the treatment of inflammatory bowel disease [8]. 

Recently, results have clearly indicated that the anti-inflammatory activity of verbascoside in a dinitrobenzenesulfonic acid (DNBS)-induced inflammatory bowel disease model can be enhanced by peroxisome proliferator-activated receptor (PPAR)-*α*. These observations suggest that verbascoside could use the same pathway as PPAR-*α* agonists in inflammatory diseases [11].

Based on these interesting data, and in continuation of our efforts toward studying this interesting molecule [12], we started the current investigation with the objective of verifying the behavior of the active ingredient in solution or in dermocosmetic preparations, in view of its potential use in cosmetics. Stability studies were performed by HPLC and took into consideration solutions at different pH value and storage conditions or cosmetic formulations containing the active. The antioxidant activity was determined by a PCL assay. Despite the good antioxidant activity measured, the highly hydrophilic character of the active ingredient was envisaged by us as a limit in its application in different types of formulations. In order to overcome this limitation, we decided to attempt simple chemical modifications of the molecule in order to make it more lipid-soluble and thus incorporable into the lipophilic phase of the cosmetic formulations, which represents a more stable matrix. This approach, usually undertaken to increase the stabilization of water-soluble ingredients, has been successfully applied to unstable products such are ascorbic acid, tocopherol and retinol [13]. Stabilization is generally obtained by esterification, so that the newly synthesized lipophilic molecules may be converted chemically or enzymatically in their bioactive form in the site of application.

In this paper, we report the results of this experiment and the stability-activity studies carried out on VPP, obtained by semi-synthetic approach, in the effort to reduce the hydrophilic profile of parent molecule without affecting the required antioxidant activity. Finally, verbascoside itself was also enclosed in a pharmaceutical topical lipid form (suppositories), and the stability analysis was conducted with the aim to determine suitability for a possible use in the treatment of inflammation of intestinal mucosa. A preliminary investigation conducted on VPP in the same formulation showed promising results.

## 2. Results and Discussion

**Scheme 1:**
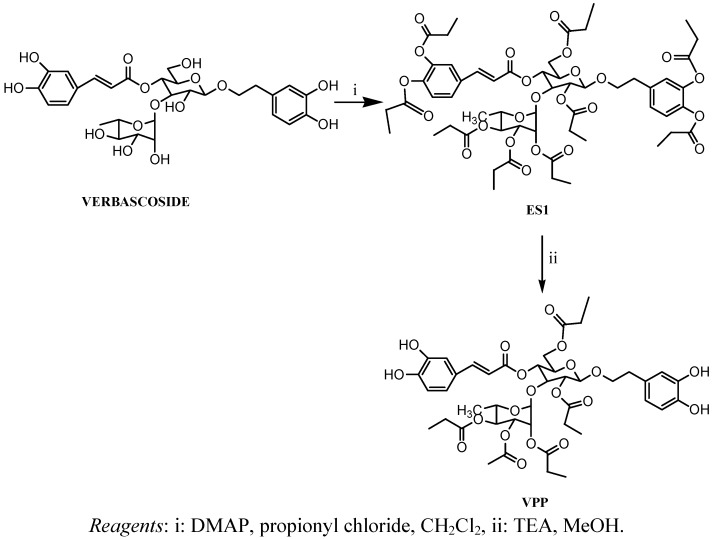
Synthesis of the pentasubstituted derivative of verbascoside (VPP).

Phenylpropanoid compounds are characterized by cinnamic and phenylethanolic moieties linked to the same glucopyranose molecule (usually glucose) by an ester and glycosidic bond, respectively [16]. The bioactive structure of these molecules which is responsible for the antioxidant [17,18], antimicrobial [19,20], anti-inflammatory [7,9,10] and antimutagenic activities [21] is thought to be the aglycone. Verbascoside, by virtue of its chemical structure, is highly soluble in hydrophilic media. However, due to the presence in solution of radical forms of oxygen or nitrogen, its hydroxyl groups easily participate in oxidation processes that in turn lead to new species with loss of the UV absorption at a wavelength of 330 nm characteristic of catechol, hydroxytyrosol and caffeic acid. Taking all this into consideration, we have decided to modify verbascoside’s structure in order to obtain a more lipophilic molecule, with the aim of increasing the range of applications, and then to evaluate its stability and anti-oxidant properties in some topical formulations. Thus, all the hydroxyl moieties in verbascoside were esterified using an acyl chloride in the presence of 4-dimethylaminopyridine (4-DMAP) and subsequently selectively deprotected to phenolic hydroxyls, using *N,N,N*-triethylamine (TEA) in methanol to afford VPP (Scheme 1).

We first conducted a stability study with the aim of evaluating the behaviour of verbascoside and VPP as a function of time, pH and of the storage conditions. Verbascoside was analyzed in EtOH-H_2_O solution (80:20, v/v) and in buffer solutions at pH 7, 6 and 5. All the solutions were checked at room temperature kept in the dark and in oven subjected to accelerated aging at 40 °C. The results show that verbascoside in buffered solution at pH 7, after just three weeks of preservation at room temperature and two weeks in oven was completely degraded, while in the solution at pH 6 its degradation is complete in 60 days.

**Figure 2:**
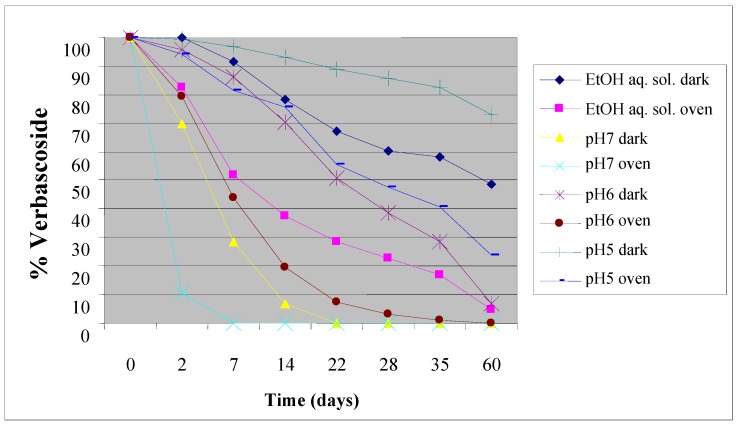
Verbascoside stability in solution at different pH value and storage conditions.

The sample at pH 5 kept in the dark showed the best verbascoside recovery percentages over time (73%), confirming its greater stability in a weak acid medium. Verbascoside also presents quite good stability in EtOH-H_2_O solution (80:20, v/v) at room temperature (Figure 2). 

Regarding VPP, we have estimated its behaviour in EtOH-H_2_O solution (80:20 v/v) because it was not soluble in the buffer solutions used for the verbascoside study. The results show that after just one week subjected to accelerated aging, VPP is completely degraded, while at room temperature and kept in the dark its degradation is complete in 60 days (data not shown).

In order to measure the lipophilic properties of the molecules, their partition coefficient (LogP), calculated as the logarithm of the ratio of concentrations of the substance in octanol and water, was evaluated. This determination was conducted in a biphasic system of octanol (20 mL) and water (20 mL to pH 6) at 25 °C. After 30 minutes of stirring, the biphasic mixture was centrifuged for 10 minutes at 4,000 rpm to complete separation of phases and then 5 mL of each phase was used for spectrophotometric determination (at 330 nm) of the concentrations of verbascoside (5 mg/20 mL) at equilibrium. A similar analysis has been done for VPP (5 mg/20 mL). The data are described in Table 1 and show a significant difference in the distribution of VPP, higher in octanol than in water, (0.96 LogP), wherease verbascoside was less concentrated in octanol than water with a LogP −0.56. These data suggest that VPP, being more lipophilic than verbascoside, accumulates in the lipid phase of the formulations thus reversing both the solubility and active formula compartment.

**Table 1:** Partition coefficients of verbascoside and VPP in a biphasic mixture of octanol and water.

	Octanol (mg/20 mL)	H_2_O (mg/20 mL)	LogP *
**Verbascoside**	1.07	3.93	−0.56
**VPP**	4.50	0.50	0.96

* LogP = log10 [mg/20 mL]octanol/[mg/20 mL]H_2_O.

**Figure 3:**
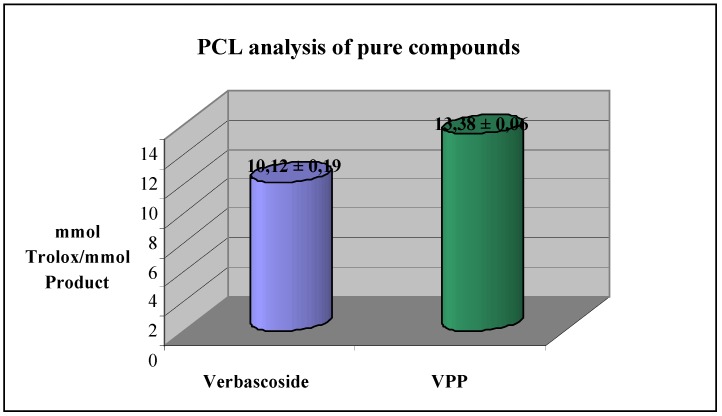
PCL analysis of verbascoside and VPP. Each value was obtained from three experiments (mean ± SE).

The two compounds were then tested to determine their antioxidant capacity by PCL analysis. Surprisingly, in the case of the semi-synthetic derivative VPP, higher antioxidant activity was observed as compared to that of verbascoside (13.38 and 10.12 mmol Trolox/mmol respectively) (Figure 3). 

Starting from this interesting data we have decided to continue the study by the investigation of the antioxidant activity and the stability of the active components incorporated into finished dermocosmetic formulations at 0.3% (p/p) and subjected to accelerated aging in oven at 40 °C.

**Figure 4:**
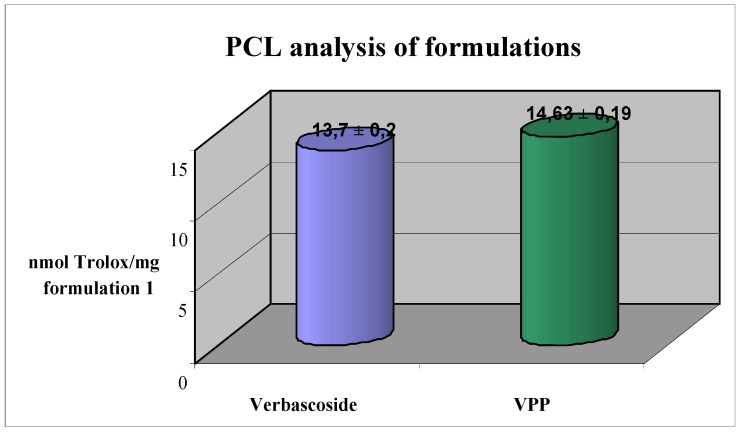
PCL analysis of formulations 1 with verbascoside and VPP. Each value was obtained from three experiments (mean ± SE).

Two different cosmetic formulations, Formulation 1 (a cosmetic base made with emollients, emulsifiers, and other ingredients that do not have any antioxidant properties) and Formulation 2 (made with water free ingredients) were employed. PCL analysis of the two cosmetic bases have confirmed the higher anti-oxidant activity of the formulation containing VPP than verbascoside (14.63 and 13.7 nanomol Trolox/mg formulation respectively) (Figure 4). PCL analysis of the same formulations over 150 days in oven have revealed a maintenance of the anti-oxidant activity (data not shown).

**Figure 5:**
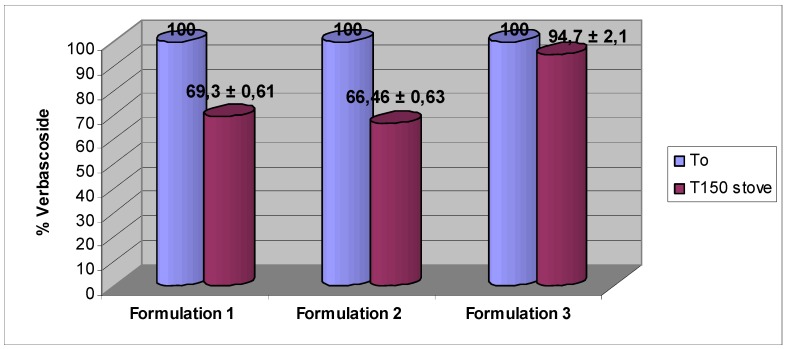
Verbascoside stability in the different formulations stored at 40 °C. Each value was obtained from three experiments (mean ± SE).

To complete the study, formulations 1 and 2 containing the active ingredients, were submitted to accelerated aging in oven at 40 °C and monitored by HPLC to evaluate the stability of verbascoside and VPP in function of time. The investigation highlighted that the concentration of verbascoside decreases by about 30% in both the formulations 1 and 2 (Figure 5) over 150 days in the oven.

**Figure 6:**
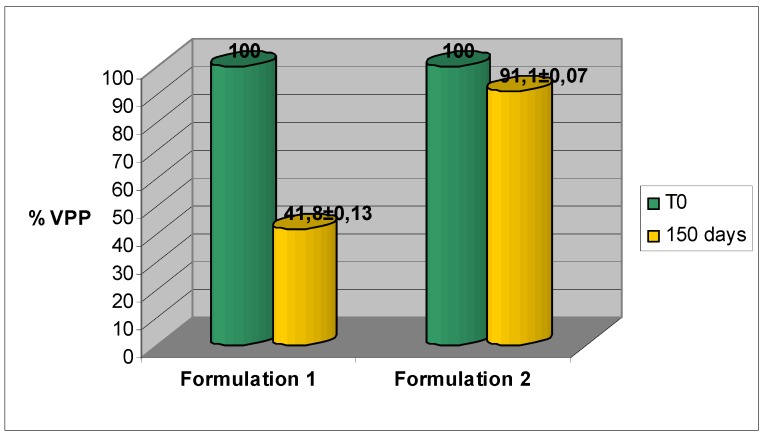
VPP recovery rate in the different formulations stored at 40 °C. Each value was obtained from three experiments (mean ± SE).

Regarding VPP, we have observed over 150 days in oven a decrease of about 60% in the formulation 1, while in the formulation 2 the active ingredient is significantly stable. In fact the decrease of VPP in this formulation is less than 10% (Figure 6).

It is well known that verbascoside has a wide spectrum of activities, including anti-inflammatory actions, so we have finally decided to incorporate the active substance in a third formulation like suppositories (0.3% p/p) with the aim to investigate its stability in a pharmaceutical lipidic form for possible future applications to the inflammation of the intestinal mucosa. We observed that after 150 days in oven at 40 °C the decrease of verbascoside was only 6% (Figure 5). A preliminary study of stability in this kind of formulation was conducted also for VPP: data show that after 30 days in oven VPP is 100% stable (data not shown).

## 3. Experimental

### 3.1. Materials and Methods

#### 3.1.1. General

All reagents were from Sigma-Aldrch srl (Milan, Italy). Reaction course was routinely monitored by thin-layer chromatography on precoated silica gel plates (Macherey-Nagel Durasil-25) by detection under a 254-nm UV lamp and/or by spraying the plates with FeCl_3_ solution or dilute potassium permanganate solution. Column chromatography was performed with Macherey-Nagel 0.063–0.2 mm/70–230 mesh silica gel. The molecular weights of the compounds were determined by ESI (Micromass ZMD 2000), and the values are expressed as [MH]^+^. ^1^H-NMR spectra were obtained using a Mercury Plus400 spectrometer. HPLC analysis was performed using an Agilent 1100 Series HPLC System equipped with a G1315A DAD and with an Hydro RP18 Sinergi 80A column (4.6 × 150 mm, 4 μm) from Phenomenex.

#### 3.1.2. Synthesis of Verbascoside Nonaproprionate (**ES1**)

To a pre-cooled (0 °C) solution of verbascoside (400 mg, 0.64 mmol) and DMAP (2.81 g, 23.04 mmol) in CH_2_Cl_2_ (40 mL), propionyl chloride (17.28 mmol) was slowly added. After stirring at room temperature for 14 h the reaction mixture was washed with H_2_O, saturated NaHCO_3_ and with Brine. The organic phase was then dried (Na_2_SO_4_), filtered and the solvent evaporated under reduced pressure. Purification of the crude residue over silica gel column chromatography (eluent: CH_2_Cl_2_/MeOH, 99/1, v/v), afforded **ES1** (72% yield) as a white foam. ^1^H-NMR (400 MHz, DMSO-*d_6_*): δ(ppm) 0.79 (t, 3H, CH_3_); 0.90–1.03 (m, 15H, 5x CH_3_); 1.09–1.15 (m, 12H, 4x CH_3_); 1.95–2.05, 2.16–2.25, 2.3–2.39 (m, 10H, 5x CH_2_); 2.55–2.60 (8H, 4x CH_2_); 2.75–2.85 (m, 2H, CH_2_-Ar, aglycone); 3.66–3.71 (m, 1H, -OCH_2_- aglycone+ 1H, sugar); 2.92–3.97 (m, 1H, -OCH_2_- aglycone +1H, sugar); 4.03–4.06 (m, 1H, sugar); 4.15–4.20 (m, 2H, sugar); 4.72–4.92 (m, 6H, sugar); 5.07–5.12 (m, 1H, sugar); 6.68 (d, 1H, *J* = 16.4 Hz; CH=CH_a_-COO-); 7.10–7.16 (m, 3H, aromatic); 7.33 (d, 1H, aromatic), 7.70–7.74 (m, 3H; 2H aromatic+ 1H Ar-CH_b_=CH); ESI MS: *m/z* 1130.1 Da [M+H]^+^, C_56_H_72_O_24_ Mol. Wt. 1129.16.

#### 3.1.3. Synthesis of Verbascoside Pentapropionate (**VPP**)

Et_3_N (2.5 mL) was added to a solution of **ES1** (518 mg, 0.46 mmol) in anhydrous MeOH (6 mL). The mixture was stirred at room temperature under argon atmosphere for 5 h and then neutralized by adding of formic acid (3 mL) at 0 °C. Next the mixture was evaporated under reduced pressure. The crude residue was dissolved in AcOEt and wash with Brine. The organic phase was than dried (Na_2_SO_4_), filtered and the solvent evaporated under reduced pressure. The residue obtained was purified by silica gel column chromatography (eluent: CH_2_Cl_2_/MeOH, 98/2, v/v), gave **VPP** (36% yield) as a white foam. ^1^H-NMR (400 MHz, DMSO-*d_6_*): δ(ppm) 0.79 (t, 3H, CH_3_); 0.92–1.02 (m, 15H, 5x CH_3_); 1.9–2.05, 2.12–2.34, (m, 10H sugar); 2.58–2.62 (m, 2H, CH_2_-Ar); 3.50–3.52 (m, 1H, -OCH_2_- aglycone); 3.65–3.75 (m, 1H, sugar); 3.85–3.9 (m, 1H, -OCH_2_- aglycone +1H sugar); 4.0 (d, 1H, sugar); 4.14–4.2 (m, 2H, sugar); 4.65 (d, 1H, sugar); 4.76–4.88 (m, 5H, sugar ); 5.03 (t, 1H, sugar); 6.21(d, 1H, *J* = 16, CH=CH_a_-COO-); 6.4 (dd, 1H, aromatic); 6.56 (d, 1H, aromatic); 6.60 (d, 1H, aromatic); 6.73 (d, 1H, aromatic); 7 (d, 1H, aromatic); 7.03 (s, 1H, aromatic); 7.51 (d, 1H, *J* = 16 Hz, Ar-CH_b_=CH); 8.65 (s, broad , 2H, 2x, OH); 9.2 (s, broad, 1H, OH); 9.65 (s, broad, 1H, OH); ESI MS: *m/z* 891.6 Da [M+H]^+^, C_43_H_54_O_20_ Mol. Wt. 890.88.

### 3.2. Antioxidant Activity Assays

#### 3.2.1. Photochemiluminescence (PCL) Method

The PCL assay, based on the methodology of Popov and Lewin [14], was used to measure the antioxidant activity of extracts with a Photochem^®^ apparatus (Analytik Jena, Leipzig, Germany) against superoxide anion radicals generated from luminol, a photo-sensitizer, when exposed to UV light (Double Bore^®^ phosphorus lamp, output 351 nm, 3 mWatt/cm^2^). The antioxidant activity was measured using both ACW (Antioxidant Capacity of Water-soluble substance) and ACL (Antioxidant Capacity of Liposoluble substance) kits provided by the manufacturer, designed to measure the antioxidant activity of hydrophilic and lipophilic compounds, respectively [15]. For ACW studies, the luminol reagent and Trolox work solution were freshly prepared according to the ACW protocol. The presence of Trolox (or any other antioxidants from the extracts) retarded luminescence for a period: hence, a lag time was noted before a signal was measured. The duration of the lag, which is calculated by the computer software from the first derivative of the detector signal at its turning point and intersection with the x-axis, was plotted against the concentration of Trolox added to the assay medium. The concentration of the added extract solution was such that the generated luminescence fell within the limits of the standard curve. Therefore, the lag time (seconds) for the ACW assay was used as the radical scavenging activity and the antioxidant capacity calculated by comparison with a Trolox standard curve and then expressed as micromoles of Trolox per gram of dry matter of red fibre. In ACL studies, the kinetic light emission curve, which exhibits no lag phase, was monitored for 180 s and expressed as micromoles of Trolox per gram of dry matter. The areas under the curves were calculated using the PCLsoft control and analysis software. As greater concentrations of Trolox working solutions were added to the assay medium, a marked reduction in the magnitude of the PCL signal and hence the area calculated from the integral was observed. This inhibition was used as a parameter for quantification and related to the decrease in the integral of PCL intensities caused by varying concentrations of Trolox. The observed inhibition of the signal was plotted against the concentration of Trolox added to the assay medium. The concentration of the added extract solution was such that the generated luminescence during the 180 s sampling interval fell within the limits of the standard curve. The extracts for ACW and ACL measurements were centrifuged (5 min at 16,000 g) prior to analysis. The antioxidant assay was carried out in triplicate for each sample, and 20 μL of the diluted extract (1:40, v/v) in HPLC-grade water (ACW) or HPLC-grade methanol (ACL) was sufficient to correspond to the standard curve.

### 3.3. Stability Studies

#### 3.3.1. Preparation of Solutions

The samples were prepared using verbascoside and VPP in different aqueous solutions. Verbascoside was solubilized in EtOH/H_2_O (80:20) and in buffered aqueous solutions with the following pH values: 5, 6, 7. VPP was only solubilized in the EtOH/H_2_O mixture. The samples have been divided in two separate series, called “dark” (naturally preserved in the absence of light.) and “oven” (subjected to accelerated aging in an oven at 40 °C).

#### 3.3.2. Cosmetic Matrices

In this study, verbascoside and VPP in different formulations have been subjected to accelerated aging in an oven at 40 °C, and analyzed by HPLC with the aim to assess their content over time. The study was carried out on three different formulations, containing 0.3% of the active ingredient. The formulations tested are as follows:

**1.** INCI: aqua, glycerin, glyceryl stearate, Ceteareth-20, Ceteareth-12, cetyl palmitate, cetearyl alcohol, dimethicone, caprylic/capric triglyceride, dicapryl carbonate, Symdiol 68T (as preservative agent for VPP formulations) or EUXYLPE9010 (as preservative agent for verbascoside formulations). From a technological point of view, it is an O/A cosmetic formulation.

**2.** INCI: Nomcort T.I.O., Nomcort HK-G, Kester Wax K82D, Covabead LH170, denaturated EtOH (for VPP) or water (for verbascoside). From a technological point of view it’s a cosmetic formulation that doesn’t contain silicone.

**3.** INCI: DubPPD1 (hydrogenated palm kernel glycerides, hydrogenated palm glycerides). From a technological point of view it’s a pharmaceutical formulation like that used in suppositories. 

#### 3.3.3. HPLC Methods

The mobile phase consisted of water/acetonitrile (95:5, 0.01 M H_3_PO_4_) (solvent A) and acetonitrile/water (95:5, 0.01 M H_3_PO_4_) (solvent B).

(1) Verbascoside: in this study, verbascoside in solution and in formulations has been subjected to accelerated aging in an oven at 40 °C, and then analyzed by HPLC with the aim to assess the content of this active ingredient over time. The determination is carried out under isocratic conditions (solvent A: 83%/solvent B: 17%). Separation was monitored with absorbance detection at 330 ± 8 nm. The flow rate was 1.0 mL/min, the injection volume was 5 μL and all separations were performed at 27 °C. 

(2) VPP: in this study, VPP in solution and in formulations has been subjected to accelerated aging in an oven at 40 °C, and analyzed by HPLC with the aim to assess the content of this active ingredient over time. The determination was carried out under isocratic conditions (solvent A: 45%/solvent B: 55%). Separation was monitored with absorbance detection at 338 ± 8 nm. The flow rate was 1.2 mL/min, the injection volume was 5 μL and all separations were performed at 27 °C.

#### 3.3.4. Statistical Evaluations

Relative standard deviations and statistical significance (Student’s t test; P ≤ 0.05) were given where appropriate for all data collected. One-way ANOVA and LSD *post hoc* Tukey’s honest significant difference test were used for comparing the bioactive effects of different samples. All computations were made using the statistical software STATISTICA 6.0 (StatSoft Italia srl).

## 4. Conclusions

The antioxidant network protects cells against oxidative injury, and when oxidative stress overwhelms this network, the subsequent modification of the cellular redox apparatus leads to an alteration of cell homeostasis leading to degenerative processes. In the dermocosmetic field, the topical application of antioxidants is often suggested as a possible strategy to prevent and modulate oxidative skin damage. We have demonstrated, in the past, that molecular combinations of different antioxidants significantly improve the efficacy of formulations where they are included [22,23]. Recently, we have investigated verbascoside as a natural model of a molecular combination (centaur approach); indeed its structure is a combination of three different moieties: two antioxidants and a sugar part. Continuing our studies on this molecule, we have prepared a possible pro-verbascoside form (VPP) by means of derivatization of the sugar hydroxyl groups but keeping free the active phenol moieties. Different formulations and pH conditions were investigated for both verbascoside and VPP. The results of the present study shows that the (water free) cosmetic formulation 2 is more stable than formulation 1 and that formulation composition and the pH values can influence in the stability of verbascoside. Moreover, the stability and antioxidant properties of verbascoside and its derivative VPP obtained by a synthetic modification in order to explore their potential as cosmeceutical and pharmaceutical ingredients should be of great interest in the dermocosmetic and medicinal fields. Our studies demonstrate the potential of this molecule which have been obtained from a sustainable biotechnology platform starting from meristemic cells.
